# Paddycrusts: interfacial bioregulators of heavy metal transport and speciation

**DOI:** 10.1128/aem.00529-26

**Published:** 2026-05-13

**Authors:** Xiaolin Kuang, Neng Ye, Junliang Xin, Yili Ge, Linsen Du, Liang Peng

**Affiliations:** 1 Research Center for Environmental Pollution Control Technology, School of Chemical and Environmental Engineering, Hunan Institute of Technology, Hengyang, China; 2 Department of Environmental Science & Engineering, Hunan Agricultural University 12575, Changsha, China; The Pennsylvania State University, University Park, Pennsylvania, USA

**Keywords:** microorganisms, cadmium, arsenic, biomineralization, bioremediation

## Abstract

Heavy metal contamination in paddy soils poses persistent challenges to food safety and sustainable agriculture. Paddy crusts (PCs) are dynamic interfacial biocomplexes that function as biogeochemical regulators of metal transport and speciation. This review aims to (i) summarize PC formation and community assembly, (ii) synthesize mechanistic processes governing metal interception, immobilization, and transformation, and (iii) evaluate evidence for PC-based remediation from laboratory systems to field trials. This review presents an integrated mechanistic framework built upon three interconnected processes: physical barrier and interception at the interface; adsorption and immobilization primarily mediated by extracellular polymeric substances (EPS) and microbial surfaces, alongside biomineralization; and redox-regulated biotransformation driving speciation shifts. We then summarize remediation performance and limitations across experimental scales, from controlled systems to field trials. The evidence underscores a generally favorable role of PCs in cadmium (Cd) immobilization and rice risk reduction. In contrast, outcomes for arsenic (As) are more variable and context-dependent. Under carbon-rich and reducing microconditions, PCs may promote Fe(III) reduction together with As(V) reduction and/or methylation, increasing As(III) and/or methylated species (e.g., DMA) in pore and surface waters. Finally, we outline future research directions, emphasizing cross-scale mechanistic understanding, synthetic community design, lifecycle risk management, and climate-smart agronomic integration. We conclude that predictable PC-based remediation requires element-specific risk screening, lifecycle management of crust maturity and senescence, and agronomic integration guided by functional indicators (EPS traits, Fe/Mn dynamics, and gene markers). These advancements are crucial to developing predictable and scalable PC-based remediation strategies for paddy ecosystems.

## INTRODUCTION

Heavy metal contamination in paddy soils poses a direct threat to global food security (1, 2). Beyond limiting crop productivity and degrading ecosystem functions, toxic metals can accumulate in rice and enter the food chain, thereby increasing human health risks (3, 4). Globally, the area of cropland affected by heavymetal contamination has been estimated at~242million hectares; among these soils,~14–17% exceed agricultural safety thresholds for at least one metal (5, 6). Cadmium (Cd) shows the highest exceedance frequency (~9.0%), with hotspots in major rice-producing regions such as southern China, northern and central India, and parts of South and Southeast Asia (5, 7, 8). By contrast, arsenic (As) exceedance is comparatively low (~1%) but spatially concentrated in southern and southwestern China and South and Southeast Asia, often overlapping with As-rich groundwater regions and thereby creating compounded exposure risks in rice-dependent populations (5, 9). Conventional physicochemical remediation approaches are frequently constrained by high costs and potential disruption to soil structure and ecological functions, underscoring the need for green and sustainable bioremediation strategies (10, 11).

**Fig 1:**
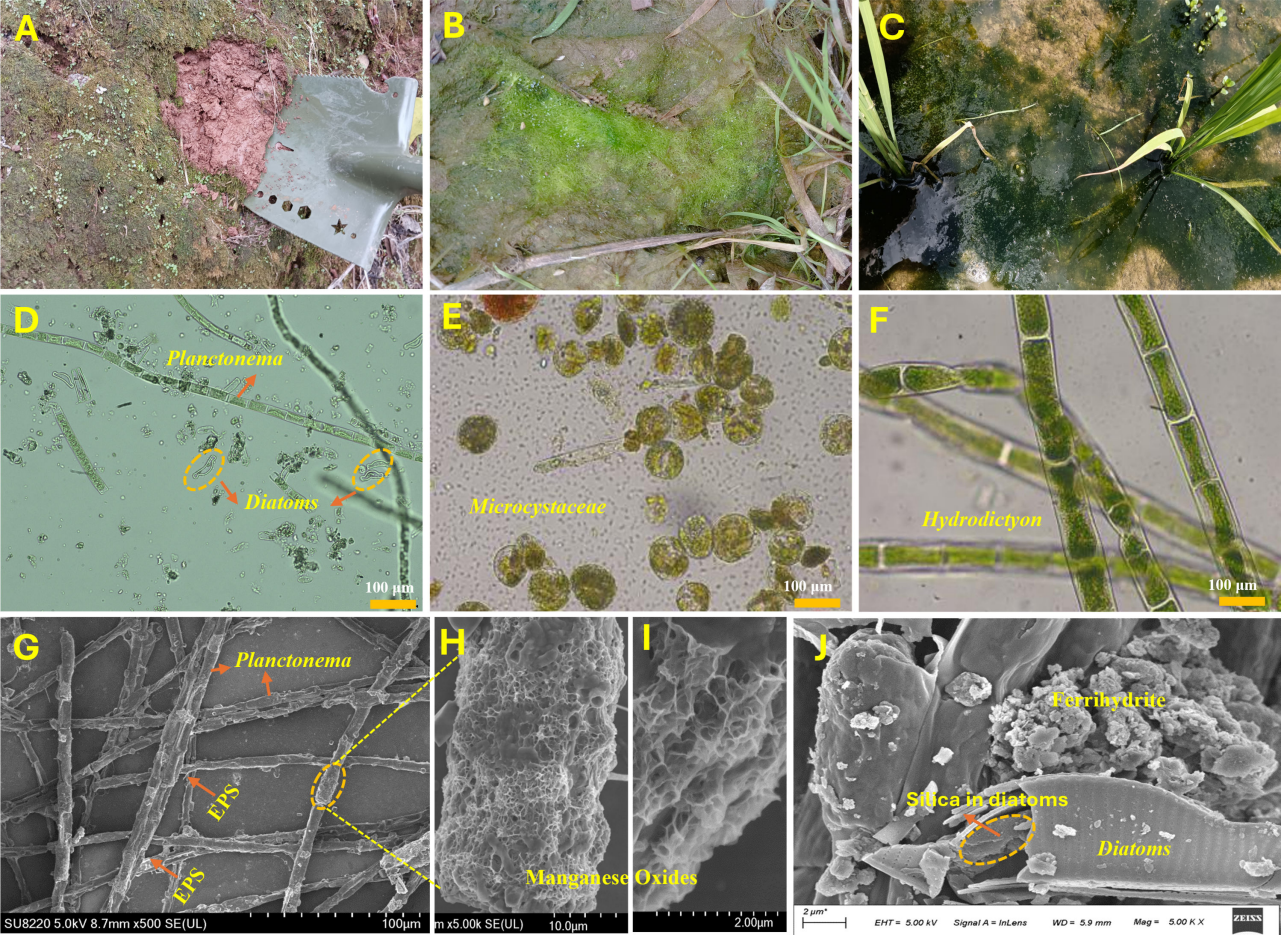
Field photographs and microscopic characterization of PCs. (**A**)Biological soil crusts in dry land. (**B and C**)PCs in paddy fields during the drying-off and flooding periods, respectively. (**D–F**) Main algal compositions observed under an optical microscope. (**G–J**) Scanning electron micrographs showing extracellular polymeric substances (EPS, **G**),manganese oxides (**H and I**),and diatoms along with other components (**J**).Panels D–Jare adapted from references 22 and 24 with permission ofthe publisher.

Biological soil crusts (BSCs) are surface biocomplexes formed by intimate associations between microorganisms (e.g., cyanobacteria, green algae, diatoms, bacteria, and fungi) and soil particles (12–14). Owing to their environmental tolerance, BSCs colonize diverse habitats ranging from extreme natural ecosystems (e.g., deserts and glaciers) to anthropogenically disturbed landscapes (e.g., mining sites) (15, 16). While early BSC research focused mainly on arid and semi-arid systems, analogous crust-like interfacial assemblages also develop in paddy ecosystems. Here, we refer to these as paddy crusts (PCs), which function as active “biogeochemical filters” at the water–soil–air interface (17–19). This filtering capacity arises from their interfacial position and structural components, including complex microbial networks, abundant extracellular polymeric substances (EPS), and biogenic Fe/Mn (oxyhydr)oxides (20, 21). Importantly, these components enable the coupling of barrier effects, adsorption, and transformation into a coordinated process that can profoundly influence metal transport, speciation, and fate (22, 23) (Fig. 1).

The capacity of PCs to immobilize metals arises from synergistic physical, chemical, and biological processes (25–27). As a dense cover at the soil surface, PCs can reduce vertical and lateral metal transfer by limiting erosion, diffusion, and resuspension, thereby decreasing transport into the root zone and surrounding water bodies (28, 29). At the microscale, the porous matrix formed by EPS, microbial filaments/hyphae, and mineral components provides high specific surface area and abundant reactive functional groups (e.g., carboxyl and phosphate), facilitating electrostatic adsorption and complexation of metal ions (30–32). Meanwhile, PC microorganisms can modulate local pH and redox conditions and drive speciation changes of redox-sensitive elements (e.g., As and Cr) (33, 34). Through biomineralization, dissolved pollutants may further be converted into stable mineral phases, supporting long-term stabilization in the solid phase (22, 24). Collectively, these mechanisms provide a theoretical basis for considering PCs as a promising nature-based, *in situ* bioremediation strategy.

The capacity of PCs to regulate metals is inherently element-specific. In many cases, PCs promote Cd stabilization, whereas they may simultaneously enhance As mobilization under certain redox–carbonconditions—an outcome that is central to evaluating both remediation benefits and potential risks (33, 35). Compared with traditional strategies such as phytoremediation and soil amendments, a systematic synthesis of PCs as nature-based “ecological engineers” for paddy heavy metal mitigation remains limited. In this review, we (i) summarize the formation and ecological characteristics of PCs; (ii) synthesize mechanistic understanding of how PCs intercept, immobilize, transform, and (in some contexts) remobilize metals, with emphasis on Cd and As; and (iii) evaluate practical strategies (*in situ* induction and artificial construction) and evidence from solution, pot, and field studies. We further identify key constraints and propose future research directions to accelerate translation of PC-based strategies into scalable paddysoil restoration and safe utilization.

## FORMATION AND ECOLOGICAL CHARACTERISTICS OF PCs

### Formation of PCs

Paddy crusts (PCs) are interfacial biocomplexes composed of inorganic minerals, organic matter, and EPS, forming microecosystems dominated by algae/cyanobacteria, diatoms, bacteria, and fungi, and in some settings may also include bryophytes or lichen-like components (29, 36). Their development typically begins with microbial colonization of soil particles and EPS secretion, followed by sequential stages of attachment, maturation, and partial dispersal/successional renewal (37). During early attachment, adhesive substrates and organic molecular templates facilitate the transition from loose association to stable cell–particle adhesion (38). As communities establish, EPS forms a three-dimensional scaffold that evolves into a mature crust with spatial heterogeneity, embedded organic–inorganic materials, and pronounced microenvironmental gradients (39, 40). Physical disturbance and biological grazing can disrupt crust structure, triggering renewed successional cycles (41, 42).

**Fig 2:**
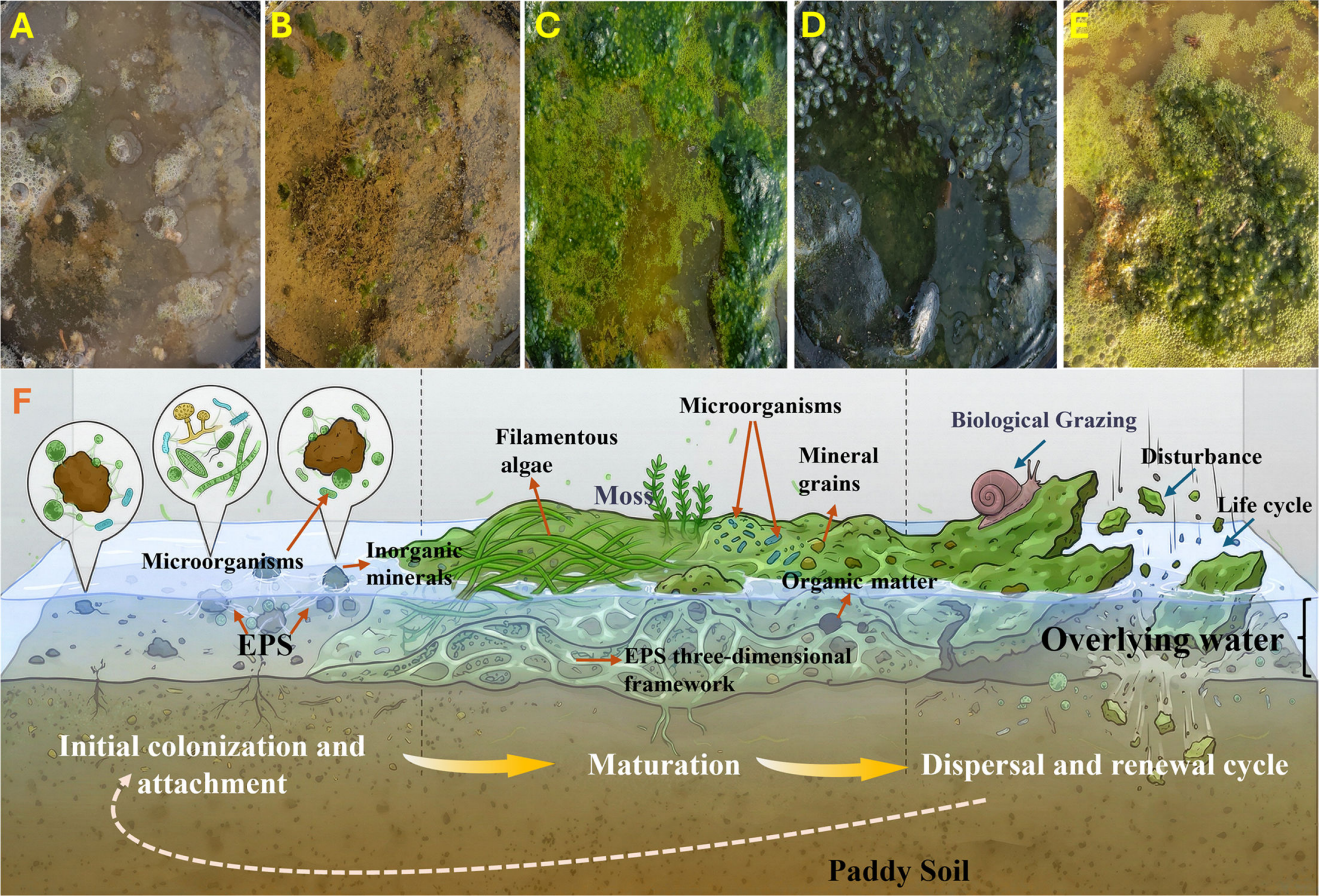
Life cycle stages of a *Microcystis*-dominated PCs (**A–E**)and a schematic diagram of PC formation (**F**).Panels C–Eare adapted from reference 20 with permission of the publisher.

Recent molecular ecological studies have advanced understanding of microbial sources and assembly in PCs (43, 44). Li et al. (45) reported that bacterial communities in periphyton were sourced almost equally from soil and overlying water (42% vs38%), whereas eukaryotic communities were derived predominantly from the overlying water (64%) rather than soil (10%). Stochastic processes (e.g., dispersal limitation) appear to dominate bacterial assembly, whereas deterministic processes (e.g., homogeneous selection) play a stronger role for eukaryotic communities. Soil ammonium-N and available phosphorus have been identified as key environmental drivers shaping PC microbial composition (46). EPS, a critical biopolymer primarily composed of polysaccharides and proteins, governs the physical stability and sorption capacity of the crusts (47, 48). Under alternating wet–dry cycles typical of paddy management (e.g., drainage), desiccation-tolerant cyanobacteria and diatoms can rapidly colonize surface soil (49). By secreting EPS, pioneer taxa establish microbial scaffolds and generate a surface layer with substantial biomass and patchy spatial distribution (12, 36, 44) (Fig. 2).

### Ecological functions of PCs

As a key interfacial component in paddy ecosystems, PCs mediate multiple ecological processes via structured microbial communities and thereby influence nutrient cycling and pollutant dynamics (50, 51). PC systems can retain and transform nitrogen (N) and phosphorus (P) in agricultural drainage water, with reported removal efficiencies of 62–90% for dissolved nitrogen and~55–99% for phosphorus, highlighting potential for non-point source pollution control (52–55). PCs can also influence carbon cycling: they may enhance organiccarbon sequestration through photoautotroph–heterotroph coupling, yet greenhousegas emissions may increase during senescence due to partial decomposition of organic matter (56–59). Structurally, the dense EPS-mediated matrix improves erosion resistance and reduces nutrient loss via runoff, providing co-benefits for soil conservation (60, 61).

Beyond nutrient regulation, PCs can affect the fate of exogenous pollutants. They may degrade certain organic contaminants (e.g., algal toxins and pesticides) via biosorption and microbial metabolism and can buffer excess nutrients in water bodies, contributing to aquatic ecological purification (62–64). Importantly, PCs exhibit strong enrichment and immobilization capacity for toxic metals (including Cd and As) and can contribute to biostabilization of Fe and Mn (22, 24, 65, 66). EPS provides abundant coordination sites that promote metal complexation and deposition (19, 67). Nevertheless, senescence-associated structural disintegration may introduce remobilization risks, emphasizing the need to consider both benefits and lifecycle constraints when applying PC-based remediation (20, 21). These coupled ecological functions create the physicochemical and biological basis for PC-driven metal interception, immobilization, and redox-sensitive transformation, which we synthesize below.

## MECHANISMS BY WHICH PCs REGULATE HEAVY METAL BEHAVIOR IN PADDY FIELDS

### Barrier and interception

PCs form a compact layer at the water–soil–air interface and can act as a primary barrier regulating metal inputs and transport. The network matrix composed of microalgae, bacteria, fungi, EPS, and microbial filaments/hyphae enhances surfacesoil stability, thereby reducing erosion induced by irrigation and rainfall and suppressing lateral dispersion and resuspension of contaminated particles (22, 28, 68). PCs can also intercept particulate and dissolved metals associated with atmospheric deposition, potentially reducing direct fluxes into the soil–water system (69). Atmospheric deposition has been reported to account for a substantial fraction of surfacesoil metal inputs in some settings (e.g., urbanized regions), suggesting that interfacial interception by PCs may contribute measurably to local metal budgets (70–72).

Unlike inert covers, PCs can buffer interfacial microenvironments through metabolic activity—for example, by regulating overlying-water pH and dissolved oxygen, and by altering pore structure and hydraulic properties of surface soil (21). These adjustments establish a more stable physical and hydrological setting for subsequent adsorption and transformation processes (73). Overall, PCs attenuate exogenous metal fluxes through physical interception and deposition trapping, while creating a semi‐closed, buffered reaction zone at the surface where further adsorption and biotransformation can take place (62). Beyond physical interception, the immobilization efficacy of PCs is further amplified by a suite of adsorption and chemical fixation mechanisms.

### Adsorption and immobilization

Building on barrier effects, PCs can immobilize metals via multi-level adsorption and fixation. The three-dimensional porous matrix formed by EPS and microbial filaments/hyphae provides a high surface area and abundant adhesion sites, enabling capture of metal-bearing fine particles through surface adhesion and mechanical entrapment (22). Wang et al. (16) reported strong interception of metal-rich particles in flowing water, resulting in reduced metal concentrations in the water column. Field observations indicate substantial enrichment of metals in PCs; for example, Cd concentrations can reach 360 mg kg⁻¹ in crusts compared with~50mg kg⁻¹ in underlying soils, while As concentrations ranging from 7.0 to 1488.9 mg kg⁻¹ have been reported. Bioconcentration factors often exceed one across sites (21, 33). Removal of EPS produces thinner, structurally weakened crusts and markedly decreases Cd sorption, underscoring the essential role of the EPS framework in enrichment and immobilization (19).

Chemical immobilization in PCs can be conceptualized as three concurrent pathways: (i) EPS-mediated adsorption/complexation, (ii) cell-surface sorption, and (iii) intracellular uptake/accumulation (30). EPS functional groups (e.g.,carboxyl, hydroxyl, and amino groups) can deprotonate near neutral pH and mediate electrostatic attraction, ion exchange, complexation, and surface precipitation (67, 74). EPS in PCs is not a uniform matrix but a chemically diverse polymeric network dominated by polysaccharides and proteins, often enriched in uronic acids and other acidic moieties that provide high densities of deprotonatable functional groups (75). Carboxyl, hydroxyl, phosphoryl, and amino groups collectively enable multiple binding modes, including electrostatic attraction, ion exchange, inner-sphere complexation, and nucleation of surface precipitates (19).

In addition to providing sorption sites, EPS can act as a diffusion-modulating gel that increases the residence time of dissolved metals and promotes co-localization of metals with reactive Fe/Mn (oxyhydr)oxide nanoparticles, thereby strengthening co-precipitation and aggregation-driven immobilization (75, 76). Importantly, EPS properties are dynamic across wet–dry cycles and lifecycle stages. Freshly produced EPS generally enhances sorption capacity and structural stability, whereas senescence and partial degradation can increase labile dissolved organic carbon (DOC), potentially stimulating reductive dissolution of Fe oxides and altering As speciation and mobility (56, 77, 78). This lifecycle dependence provides a mechanistic explanation for the shift from immobilization to remobilization observed in some PC systems and highlights EPS traits as potential functional indicators for PC management.

Potentiometric titration of PC-derived EPS reported a total functional-group site concentration of 3.9 × 10⁻² mmol g⁻¹, with polysaccharides contributing more strongly than proteins to Cd adsorption, and spectroscopic analyses identified carboxyl and amide groups as major Cd coordination sites (79). In parallel, microbial cell walls contain reactive groups that form stable surface complexes with metal ions via electrostatic interactions and ion exchange processes (80). For example, negatively charged surface groups in *Pseudomonas aeruginosa* can accumulate Pb, Cu, and Cd. Phosphate groups in Gram-negative outer membranes and carboxyl groups in Gram-positive peptidoglycan layers constitute key binding sites (81, 82).

Over longer timescales, microbially driven biomineralization can enhance long-term stabilization (83). Manganese-oxidizing bacteria catalyze Mn²^+^ oxidation and formation of poorly crystalline Mn minerals (e.g., birnessite), into whose structure Cd²^+^ may be incorporated during crystallization (14). Under optimized fertilization regimes, the fraction of Cd associated with Fe–Mn oxides can increase to approximately 55%, consistent with a transition from labile pools to mineral-bound phases (24). Microbial-induced phosphate precipitation can also immobilize Pb effectively; for example, fluidized-bed biofilm reactors have achieved>80%Pb removal within 3 h, enabled by stable biofilms (84). In PCs formed in acid mine drainage environments, diatoms may sequester Cd, Pb, and As through biosilicification, thereby trapping metals within silica frustules (22). Consistent with aquatic biofilm studies, exchangeable Pb in EPS may represent only~9% of the total Pb, with most Pb associated with Fe–Mn oxide minerals (30). Overall, rapid biosorption and slower biomineralization can act sequentially, transferring metals from labile surface-complexed states into structurally fixed mineral phases.

### Biotransformation and regulatory processes

**Fig 3:**
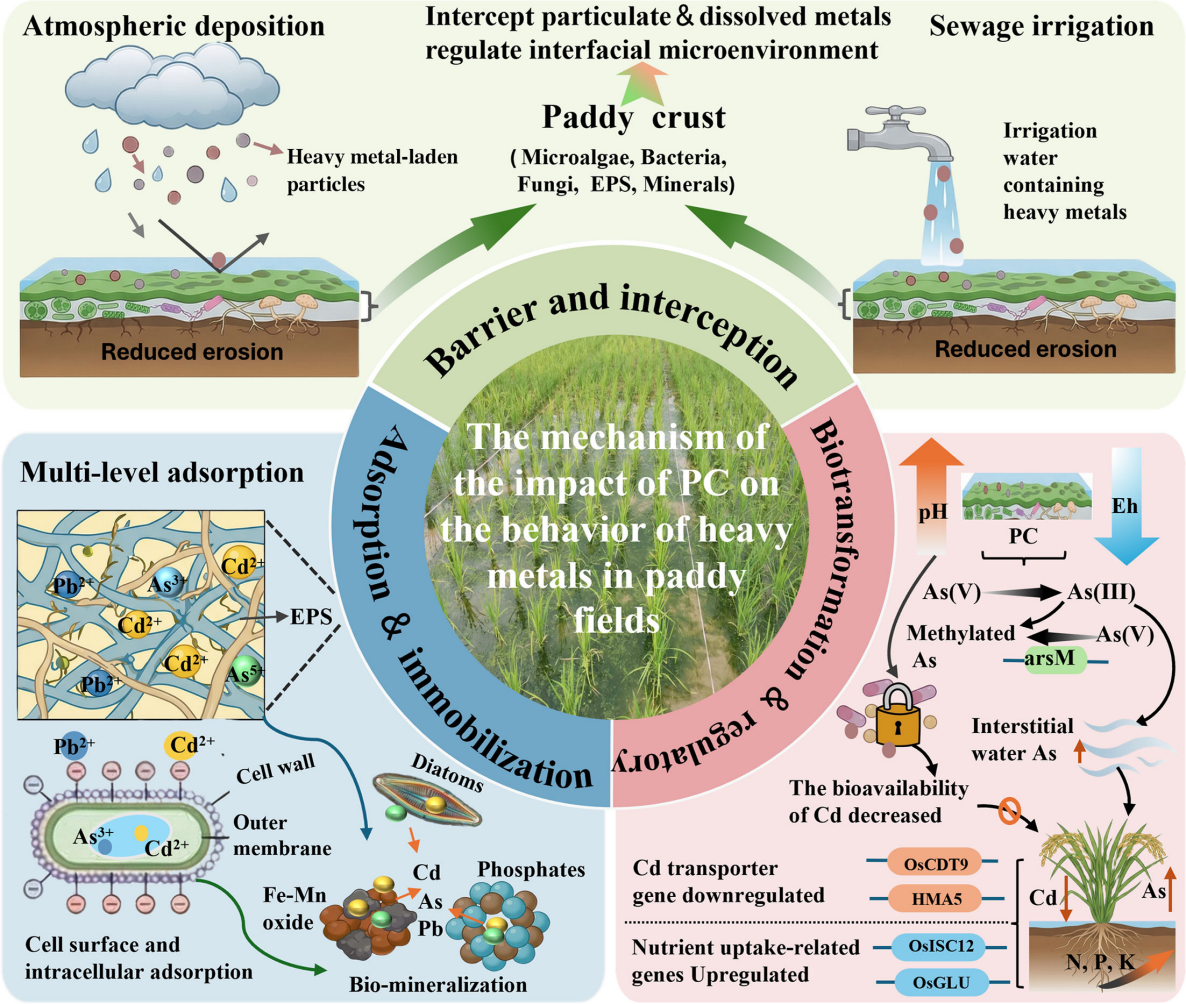
Schematic diagram of the main mechanism for heavy metal immobilization by PCs.

PCs regulate metal geochemistry and bioavailability through coupled physicochemical shifts and microbially mediated transformations. At the interfacial scale, PCs can increase overlying-water pH from~7.0 to 7.3–8.2 and elevate dissolved oxygen (20, 21). Meanwhile, within the underlying soil/PC matrix, enhanced organiccarbon inputs and microbial respiration can drive more reducing microenvironments, shifting soil redox potential (Eh) from approximately −40 to −50 mV to ~−80 to −180 mV (20, 21). These coupled changes often favor transformation of Cd from exchangeable pools into carbonate- and Fe–Mn oxide-bound forms, thereby reducing mobility and phytoavailability (24, 65). In contrast, increases in dissolved organic carbon and reductive dissolution of Fe oxides may accelerate reduction of As(V) to As(III) and enhance arsenic methylation, increasing total As and dimethylarsinic acid (DMA) in pore and surface waters (33, 35, 66, 77). Therefore, PCs can stabilize Cd while concurrently enhancing As mobilization under specific redox–carbon conditions, producing element-specific outcomes that must be explicitly considered in remediation design (35) (Fig. 3).

#### Functional genes linking microbial activity to As and Cd transformation pathways

Microbial functional genes provide a mechanistic bridge between community shifts and observed speciation patterns. PCs are enriched in functional genes involved in As transformation, notably the arsenic methyltransferase gene *arsM*, which has been reported at higher relative abundance than in surrounding soils (36, 77, 85). This genetic potential, together with increased abundance of As-reducing genera (e.g.,*Cupriavidus*), may promote As reduction and methylation (77, 86, 87). Diverse bacteria and cyanobacteria can reduce Cd²^+^/Zn²^+^ bioavailability by converting them into low-solubility or low-toxicity forms via polyphosphate sequestration, sulfide formation, and cysteine-rich binding proteins (88–90). To make these linkages explicit and operational for diagnosis/design, we summarize representative gene families/processes and their expected impacts on As/Cd speciation and mobility in Table 1.

**TABLE 1:** Microbial and plant functional mechanisms regulating metal(loid) mobility and ecological risk in PC systems^*a*^

Target element/functional domain	Mechanism category	Gene/process	Representative microbes/hosts	Effect on mobility and ecological risk	Reference(s)
As	Biotransformation (oxidation)	*aioA*: arsenite oxidation to arsenate	*Proteobacteria*, *Actinobacteria*, *Chloroflexi*, *Planctomycetes*	Converts mobile As(III) to less mobile As(V), enhancing adsorption to Fe/Mn oxides and reducing toxicity	(91, 92)
	Biotransformation (respiratory reduction)	*arrA*: arsenate reduction	*Proteobacteria*, *Firmicutes*, *Euryarchaeota*	Reduces As(V) to more mobile As(III) under anaerobic conditions, increasing arsenic release	(93, 94)
	Biotransformation (detoxification)	*arsC*: cytoplasmic arsenate reduction	*Cupriavidus, Afipia*, *Proteobacteria*	Produces intracellular As(III) that may be exported, potentially increasing dissolved arsenic	(95, 96)
	Biotransformation (methylation)	*arsM*: arsenite methylation	*Bradyrhizobium*, *Cyanobacteria*, *Anaerolinea*	Produces methylated arsenic species (DMA/MMA), reducing toxicity but potentially increasing mobility	(77, 97)
	Biosorption/sequestration	EPS complexation and intracellular accumulation	*Proteobacteria (Rhodanobacteraceae*), *Bacteroidetes*	EPS functional groups bind arsenic and reduce bioavailability at the soil–water interface	(98)
Cd	Biosorption	EPS synthesis and extracellular binding	Diverse PC microbes (cyanobacteria, bacteria, fungi)	EPS functional groups adsorb Cd, decreasing dissolved Cd and limiting plant uptake	(99, 100)
	Biomineralization (Mn-mediated)	Microbial Mn oxidation and Cd co-precipitation	Mn-oxidizing bacteria (e.g.,*Bacillus*, *Comamonadaceae*, *Sphingomonadaceae*)	Formation of Mn oxides (e.g., birnessite) adsorbs and incorporates Cd, enhancing immobilization	(14, 24, 25)
	Biomineralization (sulfidation)	*dsrA*, *dsrB*: sulfite reductase	Sulfate-reducing bacteria	Sulfate reduction generates sulfide that precipitates Cd as insoluble CdS	(101)
	Plant regulation	*OsCDT9*, *LCT*, *HMA5* (down-regulated)	Rice (*Oryza sativa*)	Reduces Cd uptake and root-to-shoot translocation	(102)
	Intracellular sequestration	*HMA9* (up-regulated)	Rice (*Oryza sativa*)	Enhances vacuolar Cd sequestration and reduces toxicity	(102)
Mn-mediated processes	Oxidative biomineralization	Microbial Mn oxidation	Mn-oxidizing bacteria	Produces Mn oxides that adsorb and immobilize trace metals	(23, 24)
Plant nutrient response	Nutrient transporter regulation	*OsNRT2.4*, *PTR9*, *PHF1*, *PT8*, *HAK1*, *HAK7*, *AKT2*, *OsISC12*, *OsGLU*	Rice (*Oryza sativa*)	Enhanced nutrient acquisition under Cd stress improves plant tolerance and indirectly reduces Cd toxicity	(102)

^*a*^ Gene functions summarized here are derived from studies of periphytic biofilms, paddy soil microbiomes, and broader environmental microbiology literature. Direct genomic evidence from paddy crust systems remains limited for some genes.

#### Community composition controls microzones and thus speciation trajectories

PCs are not functionally uniform; dominance by cyanobacteria, diatoms, or heterotrophic reducers can shift microscalepH–Eh–DOC landscapes and produce divergent outcomes. Cyanobacteria-dominated PCs tend to generate strong diel oxygenation and pH elevation via photosynthesis in the surface layer, which can enhance Fe(II) oxidation and the formation of reactive Fe(III) (oxyhydr)oxides, thereby favoring sorption/immobilization of Cd and As(V) (20, 103). However, cyanobacterial biomass and EPS can also provide labile carbon upon senescence, potentially stimulating Fe(III) reduction and releasing sorbed As, especially under stagnant flooding (33, 52). Diatom-rich PCs contribute silica frustules and often associate with Fe/Mn coatings, potentially favoring co-precipitation and structural entrapment of metals (22); diatom-derived EPS chemistry may further strengthen complexation (22, 65). In contrast, heterotrophic reducer-enriched PCs can intensify reducing microzones, accelerating Fe oxide dissolution, As(V) reduction, and, in some contexts, As methylation, thereby increasing dissolved As(III)/DMA (33, 104).

From a mobility perspective, As mobilization is favored when arsenate-reducing and methylating populations are active under reducing, carbon-enriched niches, increasing the proportions of As(III) and methylated species (e.g., DMA),which are typically more mobile than strongly sorbing As(V) under oxic conditions (52, 77). Conversely, when phototroph-driven oxygenation and Mnoxidation create oxidizing microzones, As(III) can be oxidized back to As(V), enhancing retention via adsorption to Fe(III) oxides (105–107). Therefore, the apparent “mobility” of As in PC-treated systems reflects the balance between (i) Fe/Mn oxide stability and sorption capacity and (ii) biological conversion pathways that shift As toward more weakly sorbing and thus more mobile forms.

#### Plant-level regulation and design implications

At the plant level, transcriptomic analyses indicate that PC treatment can downregulate Cd uptake-related genes (e.g.,*OsCDT9*, *LCT*, *HMA5*) and upregulate nutrient uptake-related genes (e.g.,*OsISC12*, *OsGLU*, *OsHAK7*), thereby limiting Cd accumulation and enhancing nutrient acquisition in rice (108). Collectively, by jointly regulating interfacial pH–Eh–DOC conditions, microbial functional potential, and plant transcriptional responses, PCs can drive element-specific transformations and control of metals such as Cd and As (Fig. 3). Practically, PCs are most predictable for Cd when EPS production and Fe/Mn oxide stabilization are maintained and when farming practices avoid prolonged strongly reducing conditions. For As-risk soils, performance should be screened with functional indicators (e.g., arsM and reductive markers) and managed via water–nutrient co-optimization to prevent sustained reductive dissolution and excessive methylation (33).

Taken together, current evidence indicates that the regulatory effects of PCs on heavy metals may arise not from a single mechanism but from the coupled interactions among microbial metabolism, EPS-mediated sorption, and redox-driven mineral transformations at the soil–water interface (22, 35, 106). These processes generate strong but element-specific outcomes, in which Cd is typically stabilized through adsorption and mineral association, whereas As mobility depends more sensitively on redox conditions, organic carbon availability, and microbial transformation pathways (65, 104). Consequently, predicting PC-mediated remediation requires integrating community composition, microscale geochemical gradients, and crust lifecycle dynamics rather than considering individual processes in isolation. Such a mechanism-oriented perspective provides an important foundation for designing more predictable and adaptive PC-based remediation strategies in paddy ecosystems.

## PRACTICAL APPLICATIONS OF PCs FOR REMEDIATING HEAVYMETAL CONTAMINATION IN PADDY FIELDS

### *In situ* induction and artificial construction

Effective PC-based remediation relies on two strategies: *in situ* induction and artificial construction. *In situ* induction promotes natural colonization and succession of indigenous interfacial communities by optimizing water management and nutrient supply (77). Under suitable field conditions, PC biomass has been reported to increase from~70 to 520 g m⁻² within~50 days, forming a continuous functional layer that supports metal sorption, immobilization, and transformation (29, 109). This approach avoids introducing exogenous inocula and may be particularly suitable for moderately contaminated fields where native microbiota remain functional (24).

In more severely contaminated or disturbed fields, artificial construction may be required to establish functional PCs within a practical timeframe. Stress-tolerant and metal-accumulating strains can be isolated from contaminated or extreme environments, formulated into mixed inocula, and applied to fields to rapidly build PCs with targeted remediation functions (79, 110). Improved understanding of natural PC organization has also enabled the rational design of synthetic crusts, assembled from selected algal and bacterial strains to enhance enrichment and immobilization of specific metals (e.g., Cd, Pb, Cu), thereby increasing flexibility across contamination scenarios (77, 110).

### Evaluating remediation performance: from laboratory studies to field validation

Evidence across experimental scales indicates that PCs can mitigate metal risks, yet outcomes are strongly dependent on metal type, crust community composition, and agronomic context (Table 2). In solution systems, PCs exhibited direct removal capacity for both Cd and As, with Cd removal reaching up to 74% at an initial concentration of 19.5 µg L⁻¹ (24). For As, removal efficiency can reach 78% at 3.5 mg L⁻¹, and PC treatment has been reported to improve early plant performance (e.g., higher germination and seedling biomass) while reducing As in roots and shoots under specific exposure conditions (98). Notably, crust origin can modulate performance: mine-originated crusts (MC) showed higher Cd removal (~44%) than conventional PCs (~30%) at 15.3 µg L⁻¹ Cd in water (79).

**TABLE 2:** Removal and fate control of cadmium and arsenic by PCs: a multi-scale evidence summary from solution to field studies

Type of experiment	Heavy metalandconcentration	Crust type	Key results	Reference
Solution	Cd in water:19.5 µg L⁻¹	PC	The highest Cd removal rate reached 74%	(24)
	As in water:1.5, 3.5 mg L⁻¹	PC	As removal efficiency was 78% at 3.5 mg L⁻¹;this treatment increased riceseed germination rate by 18% and seedling dry biomass by 103%, while decreasing As content in roots by 8–34% and in shoots by 9–48%	(98)
	Cd in water:15.3 µg L⁻¹	PC/mine-originated crust (MC)	The Cd removal rate was approximately 30% for PC and 44% for MC	(79)
Pot	Cd in soil:5, 50 mg kg⁻¹	PC	Cd accumulation in rice decreased by 74% (at 5 mg kg⁻¹) and 41% (at 50 mg kg⁻¹) compared to the control without PC	(21)
	Cd in soil:5, 50 mg kg⁻¹	PC	Grain Cd content was reduced by more than 80%	(102)
	Cd in soil:5, 10, 25 mg kg⁻¹	PC (*Microcystis* dominated)	A significant increase in Cd accumulation was observed: 25–80% in roots and 60–85% in shoots	(20)
	Cd: 0.5,1.9mgkg⁻¹;As: 53.4, 360 mg kg⁻¹	PC	A contrasting effect was found: Cd content in shoots and roots decreased by 28–33%, whereas As content increased by 40–60%;plant biomass increased by 34–58%	(35)
	As in soil:105.1 mg kg⁻¹	PC	As content in rice roots increased by 48%	(77)
Field	Cd in water:15.3 µg L⁻¹	PC/MC	Rice Cd concentration was reduced to 0.8 µg g⁻¹ (16% decrease) with PC and to 0.5 µg g⁻¹ (38% decrease) with MC	(79)
	Cd in water:19.5 µg L⁻¹	PC (with fertilizer)	Under a low-concentration fertilizer regime, rice Cd content was reduced by 36%	(24)
	Cd in soil:0.5–0.8 mg kg⁻¹	PC (suspension)	A dose-dependent reduction in grain Cd (11–81%) and an increase in yield (25–36%) were observed with increasing PC application rates	(102)

Pot experiments have generally supported robust Cd mitigation in rice (Table 2), showing reported decreases in plant Cd accumulation of 41–74% across soils containing 5–50 mg kg⁻¹ Cd, and grain Cd reductions exceeding 80% in some studies (21, 102). However, lifecycle and communitycomposition effects can reverse outcomes: *Microcystis*-dominated PCs increased Cd accumulation (25–80% in roots and60–85% in shoots) (20). Moreover, under Cd–As co-contamination, PCs may decrease Cd in roots/shoots (28–33%) while increasing As (40–60%), and under high As contamination, PCs increased root As by 48% (33, 35). These findings reinforce the element-specific “Cd stabilization versus potential As activation” pattern discussed in “Mechanisms by which PCsregulate heavy metal behavior in paddy fields,” above.

Field studies further demonstrate promise but also management sensitivity (Table 2). Reported outcomes include reductions in grain Cd (e.g.,11–81%) accompanied by yield increases (25–36%) with increasing PC application rates (102). In addition, MC may outperform PCs in some settings (Cd content in rice was reduced by 16% and 38% under PC and MC treatments, respectively), and low-fertilizer regimes have been associated with additional Cd reductions (e.g.,36%) (24, 79). Overall, current evidence supports PCs as a practical nature-based option for Cd risk mitigation, while emphasizing the need for As-risk screening, community and lifecycle management, and water–nutrient co-optimization to achieve predictable field performance.

## CHALLENGES AND PERSPECTIVES

Despite strong potential, practical deployment of PCs faces multi-dimensional challenges. A primary constraint is stability and effectiveness: PC formation and function depend strongly on environmental conditions (e.g., temperature and moisture), and crust integrity can be disrupted by routine farming practices (111–115). Remediation outcomes are also element-specific and life-cycle dependent. While PCs generally promote Cd immobilization through adsorption and mineral association, they may enhance As mobilization under certain redox–carbon conditions. In addition, crust senescence and decomposition may remobilize previously sequestered metals and pose secondary pollution risks (20, 35, 116). These contrasting outcomes highlight that the effectiveness of PC-based remediation cannot be evaluated solely by short-term immobilization performance but must consider the dynamic coupling among microbial processes, mineral transformations, and crust lifecycle stages.

Although recent studies suggest that climatic variables (e.g., accumulated temperature) and nutrient availability (C/N/P) shape the large-scale distribution and community assembly of PCs (117, 118), the mechanistic links among these drivers, PC community structure, and metal interception–immobilization–transformation processes remain insufficiently resolved. As a result, it is still difficult to predict PC performance across regions with contrasting hydrothermal regimes and agricultural practices. Addressing this gap requires integrating microscale mechanisms with landscape-scale environmental gradients, particularly by linking EPS chemistry, Fe/Mn mineral dynamics, microbial functional genes, and microscale redox partitioning to climatic variables and management regimes.

Future research should emphasize cross-scale integration and predictive frameworks. Mechanistic insights at microscales (EPS chemistry, Fe/Mn mineral dynamics, functional genes, and microscale redox partitioning) should be incorporated into models that explicitly account for climatic indicators (e.g., accumulated temperature), watermanagement strategies, and nutrient inputs. At the same time, advances in synthetic ecology offer opportunities to construct more stable and functionally targeted PC communities (119). Designing engineered or guided microbial consortia that promote beneficial processes, including EPS-mediated metal immobilization and stable Fe/Mn mineral formation, may enhance the predictability and efficiency of PC-based remediation.

Equally important is the incorporation of lifecycle risk assessment into PC management strategies. The functional performance of PCs may change substantially during formation, maturation, senescence, and disturbance phases (20). Future evaluation frameworks should therefore consider not only immobilization efficiency but also the potential for remobilization during crust degradation. Establishing standardized assessment protocols and integrating optimized PC technologies into sustainable rice cultivation systems will be essential to translate PC-based remediation from experimental studies to large-scale agricultural applications.

## CONCLUSION

PCs are dynamic interfacial biocomplexes in flooded rice systems, where phototrophs, heterotrophs, EPS, and reactive Fe and Mn phases jointly shape microenvironments and contaminant fate. By functioning as an active biogeochemical filter at the soil–water–air boundary, PCs can couple physical interception with EPS-mediated sorption, redox-regulated transformation, and mineral association processes, thereby influencing metal mobility and transfer to rice. Across current evidence, PCs most consistently support cadmium risk mitigation, whereas outcomes for arsenic are more variable and depend on local biogeochemical conditions. PC performance is strongly controlled by environmental and management drivers that determine community composition, vertical stratification, and life cycle stability, and these factors can shift outcomes from immobilization to remobilization in some settings. Progress therefore depends on moving from descriptive community patterns toward function-oriented indicators and controllable design, including standardized metrics, decision boundaries for element-specific trade-offs, and management strategies that maintain beneficial functions through formation, maturity, and senescence. Future work should integrate mechanistic experiments with cross-scale models and synthetic ecology approaches to enhance the predictability and field deployability of PC-based remediation. Overall, emerging evidence suggests that paddy crusts represent integrated microbial–mineral interface systems in which EPS chemistry, microbial metabolism, and redox-driven mineral transformations jointly determine heavy metal fate. Viewing PCs through this coupled biogeochemical framework may help shift future research from descriptive observations toward predictive and design-oriented remediation strategies.

## Data Availability

All data and materials supporting the findings of this study are available in the article. Additional data are available from the author upon reasonable request.
